# A Role for Cytoplasmic PML in Cellular Resistance to Viral Infection

**DOI:** 10.1371/journal.pone.0002277

**Published:** 2008-05-28

**Authors:** Beth A. McNally, Joanne Trgovcich, Gerd G. Maul, Yang Liu, Pan Zheng

**Affiliations:** 1 Division of Cancer Immunology, Department of Pathology, The Ohio State University Medical Center, Columbus, Ohio, United States of America; 2 Department of Surgery, Comprehensive Cancer Center, Program of Molecular Mechanism of Diseases, University of Michigan, Ann Arbor, Michigan, United States of America; 3 The Wistar Institute, Philadelphia, Pennsylvania, United States of America; Washington University, United States of America

## Abstract

PML gene was discovered as a fusion partner with retinoic acid receptor (RAR) α in the t(15:17) chromosomal translocation associated with acute promyelocytic leukemia (APL). Nuclear PML protein has been implicated in cell growth, tumor suppression, apoptosis, transcriptional regulation, chromatin remodeling, DNA repair, and anti-viral defense. The localization pattern of promyelocytic leukemia (PML) protein is drastically altered during viral infection. This alteration is traditionally viewed as a viral strategy to promote viral replication. Although multiple PML splice variants exist, we demonstrate that the ratio of a subset of cytoplasmic PML isoforms lacking exons 5 & 6 is enriched in cells exposed to herpes simplex virus-1 (HSV-1). In particular, we demonstrate that a PML isoform lacking exons 5 & 6, called PML Ib, mediates the intrinsic cellular defense against HSV-1 via the cytoplasmic sequestration of the infected cell protein (ICP) 0 of HSV-1. The results herein highlight the importance of cytoplasmic PML and call for an alternative, although not necessarily exclusive, interpretation regarding the redistribution of PML that is seen in virally infected cells.

## Introduction

Since the discovery of the PML gene as a fusion partner with retinoic acid receptor (RAR) α in the t(15:17) chromosomal translocation associated with acute promyelocytic leukemia (APL) [1]–[3], nuclear PML protein has been implicated in a number of cellular processes ranging from cell growth and tumor suppression, to apoptosis, transcriptional regulation, chromatin remodeling, DNA repair, and anti-viral defense [4]–[8]. Nuclear PML is also considered the coordinating protein of sub-nuclear structures aptly named PML Nuclear Bodies (PML NBs) or nuclear domain 10 (ND10), which number approximately 5–30 structures per cell [9], [10]. PML NBs are host to a number of proteins involved in transcription, cell cycle regulation, chromatin organization and cell survival [11], [12].

It is well established that certain viral infections influence the integrity of PML NBs and result in the dispersal of PML protein. The IE1 protein of human cytomegalovirus (HCMV), the E4-ORF3 protein of adenovirus 5, the BZLF protein of Epstein Barr virus (EBV), and the L-HDAg protein of hepatitis delta virus (HDV) have all been implicated in the disorganization of PML protein and PML NBs [13]–[19]. Additionally, the Z protein of LCMV and the P protein of rabies virus have been shown to redistribute PML to the cytoplasm [20]–[23]. PML protein has also been detected in the cytoplasm of cells infected with respiratory syncytial virus (RSV), herpes simplex type 1 (HSV-1), and HIV [22]–[25]. Since the targeted mutation of PML increases the yield of infectious viral particles [26], it is postulated that the disruption of PML NBs and the re-localization of PML protein represents a viral strategy to evade a cellular resistance mechanism. While these interpretations are plausible, the origin and significance of cytoplasmic PML during viral infection remains a largely unresolved issue.

Theoretically, cytoplasmic PML can be derived from two sources. First, nuclear PML may be relocated into cytoplasm by interacting viral proteins. Alternatively, cytoplasmic PML may be derived from a bona fide cytoplasmic PML isoform that is expressed during viral infection. It is known that the alternatively spliced PML gene can generate at least 6 major isoforms that have been categorized by their varying C-terminal ends [27]. Alternative splicing of internal exons 4, 5, and 6 further increases the number of available PML isoforms, and the loss of exon 6, which houses a nuclear localization signal (NLS), enhances the potential for cytoplasmic variants. Common to all PML splice variants is the retention of exons 1–3 which contains a RING finger domain, 2 B-Boxes, and a coiled-coil domain (RBCC) important for PML/protein interactions [27]. Although the various PML isoforms may have related functions due to the retention of the functional RBCC domain, it is coming to light that isoform-specific functions exist as in the example of PML III in centrosome amplification [28]. More recently, a cytoplasmic isoform of PML has been described as having a novel function in modulating TGFβ signaling [29], [30]. Thus, an intriguing possibility is that the apparent re-localization of PML during viral infection may, in part, reflect the expression of a cytoplasmic-specific PML isoform.

One of the best-characterized viral proteins known to disrupt PML NBs and effect PML localization is the multifunctional infected cell protein (ICP) 0 of HSV-1 (ICP0). Expression of ICP0 initiates immediately upon lytic infection and plays a key role in the transactivation of viral and cellular genes [31]. ICP0-deficient mutants are defective for growth at low multiplicities of infection in cell culture, and are attenuated and fail to efficiently reactivate from latent infection in cell culture and in animals [32]. Interestingly, depletion of PML can partially complement the defects of an ICP0-null mutant virus [33]. ICP0 localizes near or at PML NBs early in infection and appears to facilitate the dispersal of nuclear PML and the proteosome-dependent degradation of high molecular weight PML isoforms [25], [34]–[36]. Some PML isoforms, however, have been observed to be recruited to viral replication compartments, and PML has been observed in the cytoplasm following PML NB disruption indicating that not all PML is degraded immediately upon infection [37], [38] Up to this point it has been generally assumed that PML found in the cytoplasm of infected cells is derived from nuclear PML.

In this study, we characterize a unique low molecular weight isoform of PML, PML Ib, which is not only inducible by IFNγ, but is expressed in cells infected with HSV-1. We show that PML Ib localizes to the cytoplasm and demonstrate that the cytoplasmic PML Ib isoform alone is capable of promoting a cellular resistance to viral infection. Surprisingly, we find that the cytoplasmic PML Ib isoform can suppress HSV-1 replication via an ICP0-dependent mechanism. Our data indicate that PML Ib sequesters ICP0 in the cytoplasm which limits viral protein accumulation and replication.

## Results

### A Novel PML Transcript Enriched in HSV-1 Infected Cells Encodes a Cytoplasmic Form of PML

**Figure 1:**
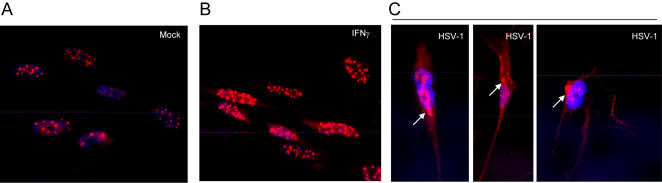
Accumulation of cytoplasmic PML in HSV-1 infected cells. Localization of PML was examined by indirect immunofluorescence microscopy in mock infected, infected (1.0 or 0.1 PFU/cell HSV-1) and IFN-γ treated MRC5 cells. Cells were fixed 24h post treatment and immunostained with anti-PML (PG-M3) and Texas- Red conjugated secondary antibody. DAPI was used to counter-stain the nucleus.

It is well established that PML protein localizes to subnuclear structures known as PML NBs. When examined by indirect immunofluorescence, the expected nuclear localization pattern of PML was observed in uninfected MRC5 cells (Fig. 1A). As has been previously demonstrated, IFNγ treatment resulted in an increase in size and number of PML NBs (Fig. 1B) [39], [40]. In contrast, a significant fraction of total PML protein localized to the cytoplasm in asynchronous MRC5 cells infected with low doses of HSV-1, as has been reported by others [37]. A careful examination of the pattern of nuclear vs cytoplasmic PML, however, suggests that cytoplasmic PML is not always formed at the expense of nuclear PML (Fig. 1C). Figure 1C left panel shows the appearance of cytoplasmic PML in a cell in which nuclear PML displays a well characterized disorganization pattern indicative of PML that has been recruited to stage IV replication compartments [37]. Accumulation of cytoplasmic PML was also seen, however, in cells with apparently normal PML NBs (Fig. 1C, middle and right panels). These observations raised the possibility that at least a portion of cytoplasmic PML seen in virally infected cells is derived from a bona fide cytoplasmic PML isoform and is not simply representative of PML that has been re-localized from the nucleus to the cytoplasm.

**Figure 2:**
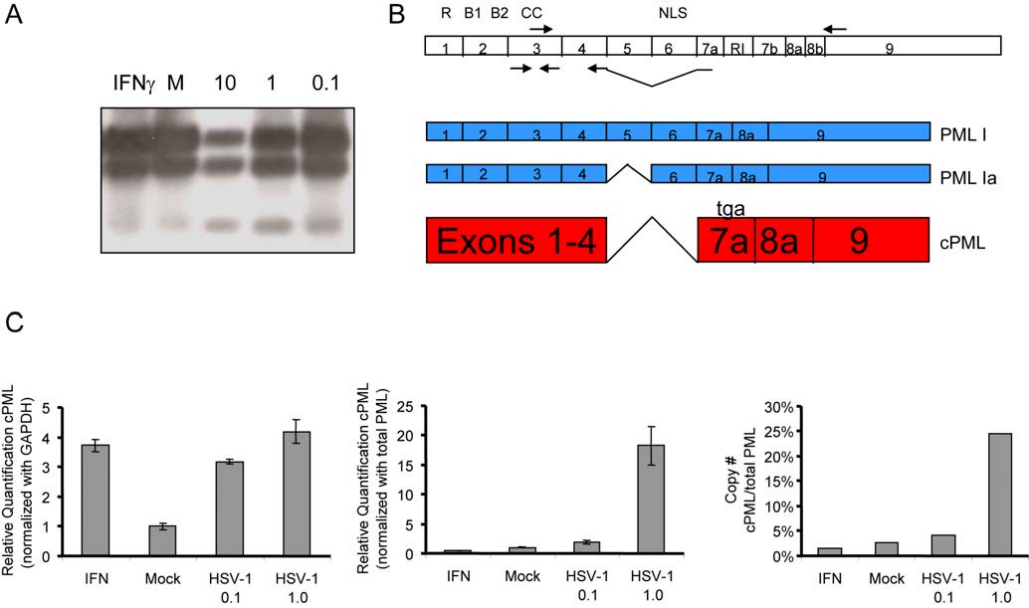
Endogenous expression of cytoplasmic PML is enhanced in virally cells. Total RNA was extracted from MRC5 cells 24 h after exposure to HSV-1 (F) at 0.1, 1.0, and/or 10 PFU/cell. Mock infected cells and cells treated with IFN-γ were used as controls. Expression of PML I transcript variants was determined by RT-PCR using a primer set located in exon 3 and exon 9 of PML. (A) Southern blot analysis using a PML cDNA probe was used to enhance signal detection (25 PCR cycles). (B) Depiction of the three PML I transcript variants identified after cloning and sequencing, and the relative locations of the primer sets. (C) RT-real time PCR was performed using a forward primer located in exon 3, and a reverse primer spanning the exon 4-7a junction of PML to specifically amplify cPMLΔ5&6 in MRC5 cells upon viral infection or IFN-γ treatment. Left panel: Relative quantification of cPML expression was normalized with GAPDH. Middle panel: Relative quantification of cPML expression was normalized with total PML using a primer set in exon 3 that amplifies all PML transcripts. Relative expression was determined using the mock sample as the calibrator. Right panel: Ratio of cPML to total PML. PML I and PML Ib standards were serially diluted to generate a STD curve containing at least 6 points. The copy number of each standard was determined using the following formula: (X g/ul DNA/[plasmid length in basepairs×660])×6.022×10 ^23^. A STD curve was generated based on a LOG10 to Ct curve. The average Ct value of each sample was used to extrapolate a Log based 10 value which was subsequently converted to molecules/ uL. These values were then used to determine the ratio of cPML Δ5&6 to total PML (results are representative of two separate trials)

To determine if viral infection could uniquely affect the expression of PML splice variants lacking an NLS, total RNA was harvested from uninfected and HSV-1 (F)-infected human primary fibroblasts (MRC5) and analyzed by Reverse Transcriptase Polymerase Chain Reaction (RT-PCR). By using a forward primer recognizing a sequence at the end of exon 3 of PML and a reverse primer recognizing a sequence at the beginning of exon 9 we were able to examine the internal exon splice variants of the PML I transcript which is believed to be the predominant PML isoform [41]. As shown in Figure 2A, multiple bands were generated. Most notable, however, was the presence of a low molecular weight band seen in HSV-1 infected cells. Cloning and subsequent sequencing of the PCR pool revealed that the fast migrating band corresponded to a sequence indicative of an isoform missing both exons 5 and 6. According to conventional nomenclature, the transcript represents the predicted PML Ib isoform [27].

Interestingly, the splicing of exons 5 and 6 in any PML isoform variant retaining exon 7a, results in a frameshift and the subsequent formation of a termination codon encountered in the beginning of exon 7a. Thus, the splicing of exons 5 and 6 from PML transcript variants I-V results in transcripts that ultimately encode the same protein (Table 1). As such, by using a forward primer in PML exon 3 and a reverse primer that spans the exon 4/7a junction (see Fig. 2B) we were able to examine the expression of several PML Ib–like transcripts. We therefore used this primer set to quantify by real time PCR the level of PML isoforms lacking exons 5 and 6 (collectively called cPMLΔ5&6) upon viral infection. In relation to the housekeeping gene, GAPDH, cPMLΔ5&6 transcripts were increased 3- to 4- fold during viral infection. A similar increase was observed after IFNγ treatment (Fig. 2C, left panel). It has long been known, however, that HSV rapidly shuts down host RNA synthesis [42]. To circumvent the challenge of ascertaining RNA expression in infected cells with waning levels of housekeeping genes, real-time PCR was performed using primers specific for the cPMLΔ5&6 transcripts along with primers that recognize all PML isoforms (primers derived from exon 3) to compare the expression of cPMLΔ5&6 to total PML transcripts during viral infection. As shown in Fig. 2C, middle panel, relative quantification of cPML showed that cPMLΔ5&6 mRNA was enriched approximately 20 fold when cells were infected at 1.0 PFU/cell. Likewise, absolute quantification analysis of cPMLΔ5&6 demonstrated that approximately 25% of total PML in virally infected cells are cPMLΔ5&6 (Fig. 2C, right panel). Given the large number of PML isoforms that are expressed in infected cells, it is clear that cPMLΔ5&6 constitutes a major fraction of PML that exists during viral infection.

**Table 1:** Predicted open reading frames of the various internal splice variants of PML: Five different PML transcripts ultimately generate the same protein product.

Isoform	Exons	ORF (bp)				Nomenclature
		Fl	Δ5	Δ56	Δ456	
PML I	1-2-3-4-5-6-7a-8a-9	2646	2502	1269	2172	TRIM19 alpha [63]; PML-1 [64]; PML4 [65]; variant1 (NCBI)
		(882aa)	(834aa)	(423aa)	(724aa)	
PML II	1-2-3-4-5-6-7a-7b	2472	2328	1269	1998	TRIM19 gamma [63]; PML-3 [64]; variant 3 (NCBI)
		(824aa)	(776aa)	(423aa)	(666aa)	
PML III	1-2-3-4-5-6-7a-7ab-7b	1923	1779	1269	1449	PML-L [2]
		(641aa)	(593aa)	(423aa)	(483aa)	
PML IV	1-2-3-4-5-6-7a-8a-8b	1899	1755	1269	1425	TRIM 19 zeta [63]; PML3 [65]; Myl [66]; variant 6 (NCBI)
		(633aa)	(585aa)	(423aa)	**(475aa)	
PML V	1-2-3-4-5-6-7a-7ab-RI	1833	1689	1269	1359	TRIM19 beta [63]; PML-2 [64]; PML1 [65]; variant 2 (NCBI)
		(611aa)	(563aa)	(423aa)	(453aa)	
PML VI	1-2-3-4-5-6-RI-7a	1680	1536	1473	1506	TRIM19 epsilon [63]; PML-1 [3]; PML-3b([64]; variant 5 (NCBI)
		(560aa)	(512aa)	*(491aa)	(502aa)	

Notes:

TRIM 19 iota & TRIM 19 eta (aka variant 7) both labeled PML VI b by Jensen [27] = PML Ib-like transcripts

* PML VI contains intron sequence GTAGGGAG before exon 7a such that a PML Ib-like isoform is not generated

** cPML isoform connected with TGF-β signaling [29]

Table adapted from Jensen et.al, 2001.

### Localization of PML Ib and its Effects on Nuclear PML

**Figure 3:**
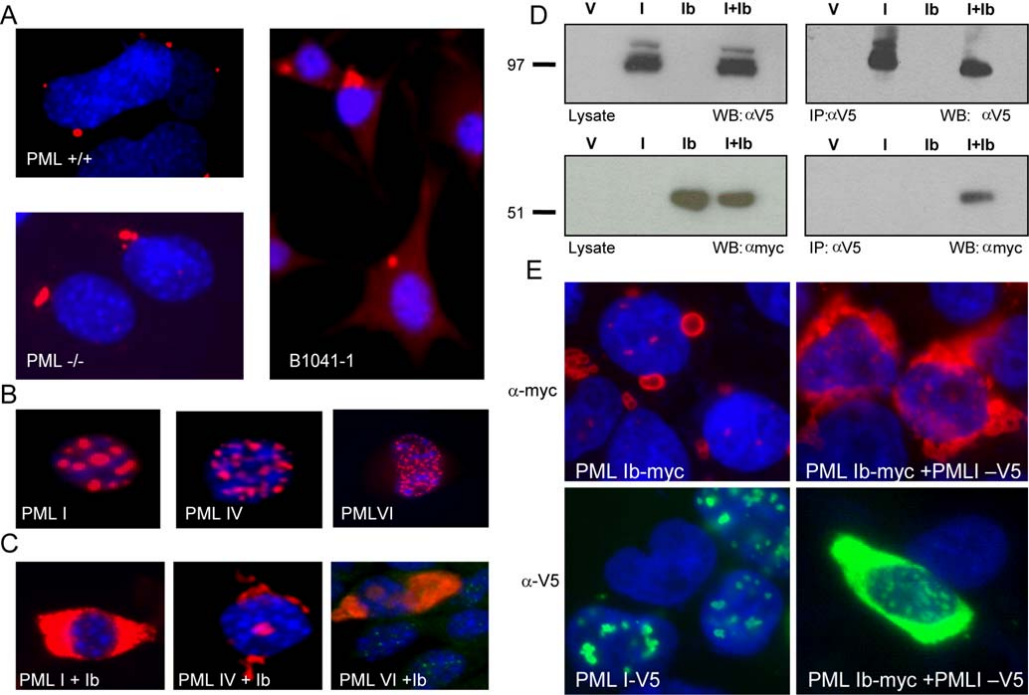
PML Ib primarily localizes to the cytoplasm and influences the localization of nuclear PML isoforms. (A) Immunolocalization of PML Ib. PML ^+/+^ PML ^−/−^, and B1041-1 mouse fibroblast cell lines transfected with a human PML Ib/pcDNA3.1 construct were immunostained with PG-M3 to determine the intracellular localization of PML Ib by confocal (PML^+/+^) and indirect (PML^−/−^ and B1041-1) immunofluorescence microscopy. DAPI was used to counter-stain the nucleus. (B) Localization of PML isoforms I, IV, and VI was revealed by immunostain with PG-M3 and Texas Red following the transfection of each cDNA into B1041-1 cells. (C) Co-transfection of PMLIb with nuclear PML isoforms induces a redistribution of PML protein to the cytoplasm: PMLIb+PML I (stable transfectant, left image); PMLIb+PML IV (transient transfection, middle image); PMLIb+PML VI (transient transfection, right image). Cells were immunostained with PG-M3 and Texas red conjugated secondary antibody as above. Note that the cells transiently co-transfected with PML Ib+PML VI (leftmost image) are dual stained with PG-M3 and a murine polyclonal PML antibody and show re-localization of endogenous mouse PML. DAPI was used to counter-stain the nucleus. Localization of PML was examined by indirect immunofluorescence microscopy. (D & E) Interaction between nuclear and cytoplasmic PML isoforms. V5-tagged PML I/ pcDNA 4, myc-tagged PML Ib/pcDNA 3.1 and vector constructs were transiently transfected either alone with vector or in combination, into the HEK293 cell line. 48h post-transfection cells were (D) lysed and subjected to co-immunoprecipitation or (E) fixed and immunostained. (D) V5 and myc antibodies were used for immunopreciptiations while HRP-conjugated V5 and myc antibodies were used for immunoblots. As shown in the bottom right panel, myc-tagged PML Ib co-precipitated with V5-tagged PMLI, whereas transfection of PML I or PML Ib alone did not result in co-immunoprecipitation. Panels on the left (top & bottom) represent immunoblots of the crude lysates (10 µg protein/lane), and are included to show the antibody specificity for the V5-tagged PML I and Myc-tagged PML Ib constructs. V = vector control, I = PML I, Ib = PML Ib, I+Ib = co-transfected PML I & PML Ib. (E) Cells co-transfected with both vectors, PML I-V5+pcDNA 3.1-myc, PML Ib-myc+pcDNA 4-V5-his, or PML I-V5+PML Ib-myc and grown on glass cover slips were single stained with a FITC conjugated V5 antibody (bottom two panels), or the anti-myc antibody, 9E10, and anti mouse Texas Red (top two panels) as indicated in the figure.

Because the nuclear localization sequence (NLS) for PML is located on exon 6 it was of interest to determine how the deletion of exons 5&6 effected the localization of PMLIb. The sub-cellular localization of PMLIb was verified by generating a human PMLIb /pcDNA 3.1 Hygro construct which was used to transfect murine PML ^+/+^ and PML ^−/−^ fibroblasts. PMLIb was then visualized using an antibody specific for human PML. Confocal analysis indicated that PMLIb localizes at or adjacent to the nuclear membrane and can assume a hollow sphere shape (Figure 3A top, left panel) Immunofluorescence microscopy of PMLIb in stably transfected in murine fibroblasts revealed a perinuclear distribution with a distinct localization of PMLIb to discrete sites in the cytoplasm regardless of whether the cell expressed endogenous PML (Fig. 3A compare PML ^+/+^ & B1041-1, to PML ^−/−^ cells bottom left panel). Thus, the cytoplasmic localization of PMLIb is not dependant on its interaction with other PML isoforms. Interestingly, however, when the PMLIb cDNA was co-transfected with cDNAs encoding predominantly nuclear localizing PML isoforms (PML I, IV, and VI, Figure 3B) the cytoplasmic PML aggregates appeared significantly larger and surrounded the entire nucleus (Fig. 3C). Furthermore, when an anti-mouse PML antibody was used to visualize endogenous PML, it became clear that the human PMLIb isoform was capable of re-localizing endogenous PML (Fig. 3C rightmost panel). To further investigate the interaction of cytoplasmic and nuclear PML isoforms a V5-epitope-tagged PML I plasmid and a myc-epitope-tagged PML Ib plasmid were constructed. The differentially tagged constructs were transiently transfected alone or combined into HEK293 cells, and cell lysates were subsequently subjected to co-immunoprecipitation with antibodies specific for the myc or V5 tags. A stable V5-PML I/Myc-PML Ib heterodimer would contain both isoforms when precipitated with the V5 antibody and blotted with the myc antibody. As shown in figure 3D (right panels, far right lanes marked I+Ib), anti-V5 co-immunoprecipitated V5-tagged PML I and myc-tagged PML Ib following transient co-transfection. As expected, V5-tagged PMLI alone could be precipitated by the anti-V5 antibody and detected by immunoblot with anti-V5 antibody, but not with the anti-myc antibody (compare top & bottom right panels, second lane marked ‘I’). Likewise, myc-tagged PML Ib alone could not be detected by immunoblot with the anti-myc antibody after immunoprecipitation with anti V5. Similar results were observed using a V5-epitope-tagged PML IV construct (data not shown). Our attempts to carry out reciprocal analysis using anti-myc to co-precipitate V5 tagged PML were unsuccessful. Nonetheless, it appears that a physical association can occur between nuclear and cytoplasmic PML isoforms. As a follow-up to the above studies, indirect immunofluorescence was used to assess the localization pattern of co-transfected PML Ib-myc tagged and PML I-V5 tagged cDNAs. As shown in figure 3E, single staining for PML Ib using the anti-myc antibody, 9E10, demonstrated that PML Ib primarily localizes to cytoplasmic dots or rings when transfected alone, but assumes a perinuclear distribution when co-transfected with PML I. Likewise, single staining for PMLI, using anti V5 antibody showed that PMLI also tends to distribute towards a perinuclear localization pattern when co-transfected with PML Ib, but is mostly found in nuclear dots when transfected alone. Although single stain analysis also revealed nuclear dot formation in co-transfected cells, anti-myc was unable to detect PML I when transfected alone and anti V5 failed to detect PML Ib when transfected alone (data not shown). Together, these findings confirm that the PMLIb isoform predominantly localizes to discrete sites in the cytoplasm and to sites near or at the nuclear membrane, and that a physical association can occur between nuclear and cytoplasmic PML isoforms that can affect the localization pattern of both isoforms.

### PMLIb as an Effector Molecule in the Cellular Antiviral Defense against HSV-1

**Figure 4:**
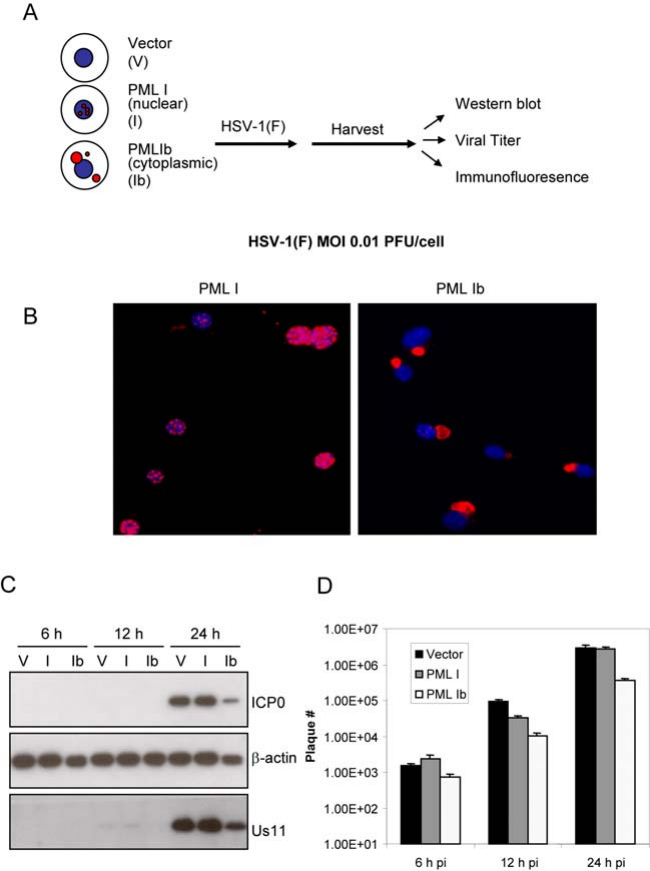
PML Ib can function in the cellular anti-viral defense against HSV-1. (A) A schema of the cellular model and infection protocol. (B) B104-1-1 cells were transfected with vector, PML I, or PML Ib. Stable cell clones were selected. PML I and PML Ib cells were immunostained with PG-M3. (C and D). Stable cell clones were infected with 0.01 PFU/mLHSV-1 -F. Infected cells were harvested at the indicated times for Western blot analysis (C), and analysis of viral tier (D). Expression of PML Ib reduced viral protein production in cells infected with HSV-1(F) (C). Protein lysates (10 µg/sample) were separated by electrophoresis and blotted with antibodies against the HSV-1 viral proteins ICP0 and US11. β-Actin was used as loading control. (Results are representative of multiple experiments.) (D) Viral titers at different time points were determined in triplicate by standard plaque assay using VERO cells.

We used stable cell lines over expressing PML I or PML Ib to examine the consequences of over-expression of nuclear PML (PML I) or cytoplasmic PML (PML Ib) on viral protein accumulation by immunoblot (diagramed in Fig. 4A). As shown in Figure 4B, after selection, the cells had uniform transfection and protein localization for nuclear PML I and cytoplasmic PML1b. The levels of both the immediate early (ICP0) and late (Us11) HSV-1 viral proteins were reduced in cells expressing PML Ib when cells were infected with HSV 1 in different time points post infection (Fig. 4C). These results demonstrate that PML Ib can impede the accumulation of both early and late viral protein levels when challenged with HSV 1. In contrast, PML I did not display anti-viral activity. Surprisingly, we consistently observed a slight enhancement of viral protein accumulation in cells transfected with PML I alone.

Concomitant with the above experiment, we titrated the material from HSV-1 infected PML I, or PML Ib -transfected cells and used it to re-infect VERO cells to determine the effect of PML isoforms on viral production. As shown in Figure 4D, ectopic expression of PML Ib resulted in an approximate 10-fold reduction in viral titer compared to vector control and PML I-expressing cells. Taken together, these results demonstrate that PML Ib limits viral protein accumulation and virus replication. In contrast, the predominantly nuclear localizing PML I isoform alone does not confer an antiviral effect.

**Figure 5:**
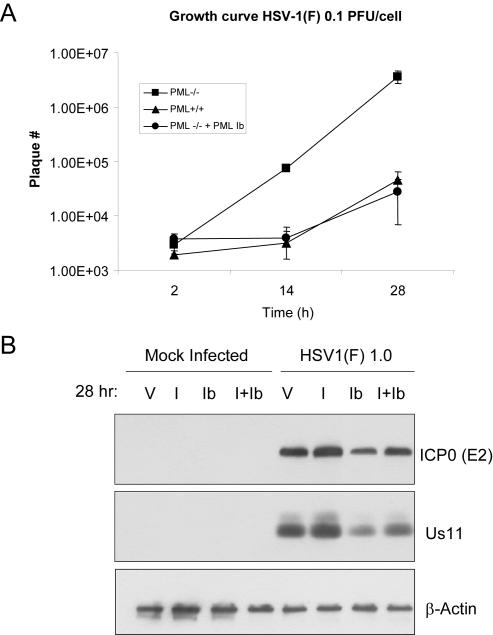
cPML exerts an antiviral effect against HSV-1. (A). PML ^−/−^, PML ^+/+^, and PML^−/−^ cells stably over-expressing PML Ib were exposed to 0.1 PFU/mL HSV-1 (F) and harvested 2, 12, and 24 h post infection. Viral titers were determined in Vero cells. (B). PML (^−/−^) cells were transfected with vector, PML I, PML Ib or co-transfected with PML I and PML Ib. Polyclonal cells were drug selected and exposed to 1.0 PFU/cell HSV-1 (F) infection. 24 h post infection, cells were harvested and Western blot analysis was performed using antibodies to ICP0, Us11, and β-Actin.

To determine whether the function of PMLIb depends on endogenous PML, PML Ib was transfected into PML^−/−^ fibroblasts and compared with PML^+/+^ and PML^−/−^ cells for their susceptibility to HSV. As shown in Fig. 5A, targeted mutation of PML caused an approximate 100-fold increase in HSV yield over a 24-hour period. Transfection of PMLIb, however, completely abrogated the increase. Thus, in this model, PMLIb is largely responsible for the anti-viral effect of the *Pml* locus.

In order to directly compare PMLI, PMLIb and PMLI+Ib for their abilities to confer resistance to viral infection, PML^−/−^ fibroblasts were transfected with either PML I and/or PML Ib and then infected with HSV-1(F). Cells were harvested 24 h post infection to compare viral protein accumulation. As shown in figure 5B, PML Ib caused a clear reduction in HSV-1 (F) protein levels. In contrast, PMLI caused a slight increase in the synthesis of viral proteins. When the two cDNA were co-transfected, PMLIb abrogated the increase caused by PMLI.

**Figure 6:**
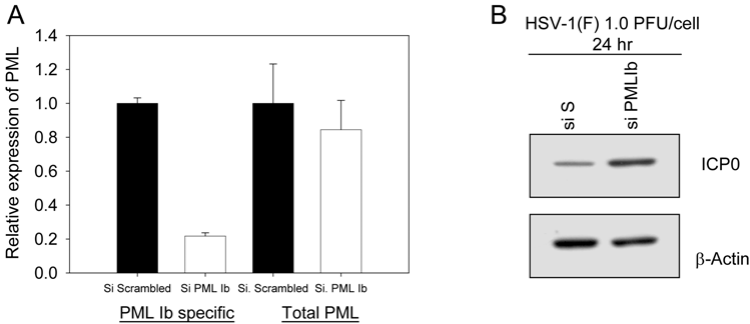
Perturbation of cPML by RNAi effects the accumulation of HSV-1 viral proteins. (A) Relative expression of the coding transcripts for cPML or total PML in MRC5 cells 96 h post nucleofection of siRNA specific for cPML (siPML Ib). Bars are grouped based on the primer sets used to detect the relative expression of cPML. Data are representative of at least two separate trials. (B). MRC5 cells nucleofected with a negative control siRNA (siS) or siRNA for cPML (PML Ib) were exposed to 1.0 PFU/cell wild type HSV-1 [HSV-1 (F)], or the ICP0 ^−/−^ HSV-1 mutant strain R7910. 24 h post infection, cell lysates were harvested for Western blot analysis of the HSV-1 viral protein ICP0. β-Actin was used as a loading control.

To evaluate the endogenous role of PML Ib and establish physiological relevance, we selectively blocked PML Ib biosynthesis in infected primary fibroblasts using a siRNA oligonucleotide specific for the junctional region of exon 4 and 7a that is found exclusively in PML Ib-like transcripts. Compared to a negative siRNA control, nucleofection of cPML Δ5&6 siRNA resulted in a more than 80% reduction in cPML Δ5&6 transcripts (Fig 6A). The specificity of the siRNA was confirmed using primers that recognize all PML isoforms. As expected, the siRNA for cPML Δ5&6 caused only a partial reduction on total PML mRNA in mock-infected cells. More importantly, knock down of PML Ib (cPML Δ5&6) resulted in a substantial increase in ICP0 viral gene expression when infected with HSV-1 (Fig. 6B). These data establish that endogenous PMLIb plays a critical role in the cellular resistance to viral infection.

### PMLIb Exerts its Antiviral Function through an ICP0-Dependent Mechanism

**Figure 7:**
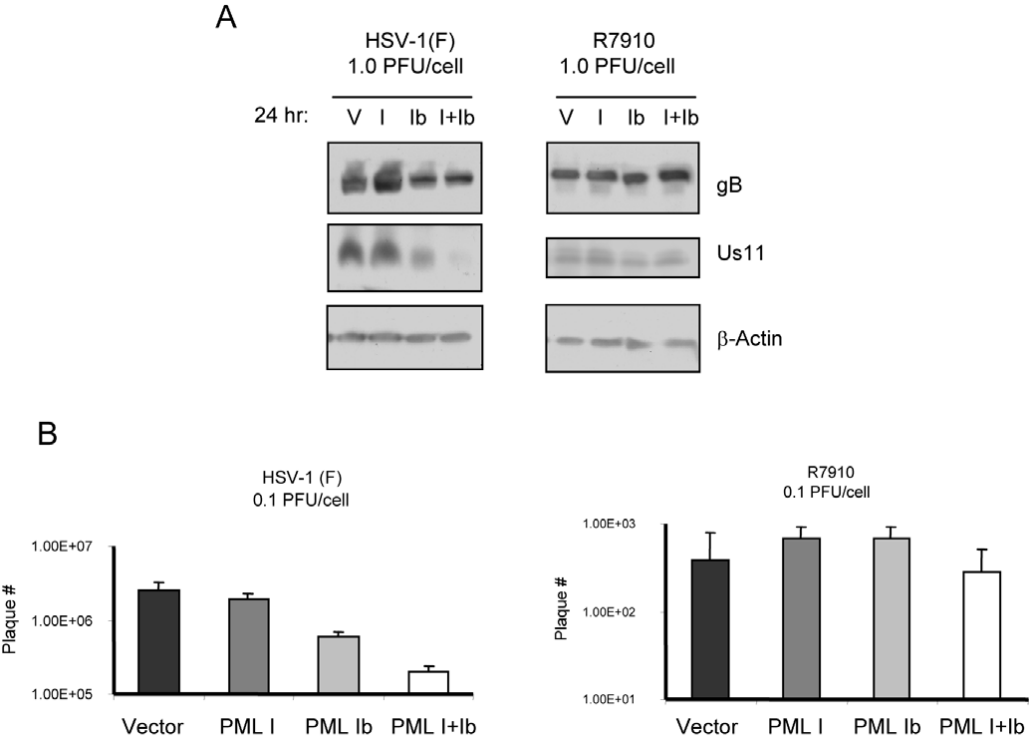
The antiviral properties of cPML are dependent on ICP0. (A) Western-blot analysis comparing viral protein expression of gB and Us11 in B1041-1 cells stably over-expressing various PML isoforms and exposed to wild type HSV-1 (F) (left panel), or the ICP0 ^−/−^ HSV-1 virus R7910 (right panel). (B) Comparison of viral growth in cells over-expressing PML and exposed to 0.1 PFU/mL of wild type HSV-1 (F), or the ICP0 ^−/−^ mutant virus R7910. Viral titers were determined in triplicate by standard plaque assay using Vero cells. P values were determined using two-sided independent sample t-tests comparing results to vector control (** P<0.001).

Previous reports have clearly established that a unique relationship exists between ICP0, PML and PML NBs in HSV-1 infected cells. Namely, ICP0 appears to migrate from the cytoplasm to target nuclear PML and PML NBs relatively early in infection [34], [36]. To determine if the antiviral properties of cPML involved ICP0, we compared the effect of PML I, PML Ib and PML I+Ib on the replication of the HSV-1 ICP0-null mutant R7910 with the WT virus HSV-1(F). While PML Ib and PML I+Ib caused a substantial reduction in the accumulation of glycoprotein B (gB) and US11 when infected with HSV-1 (F), no such effect was observed in the infection by R7910 (Fig. 7A). Likewise, inhibition of viral titer was also ICP0-dependent (Fig. 7B). These results demonstrate that ICP0 plays an essential role in the PML Ib-mediated suppression of HSV infection.

**Figure 8:**
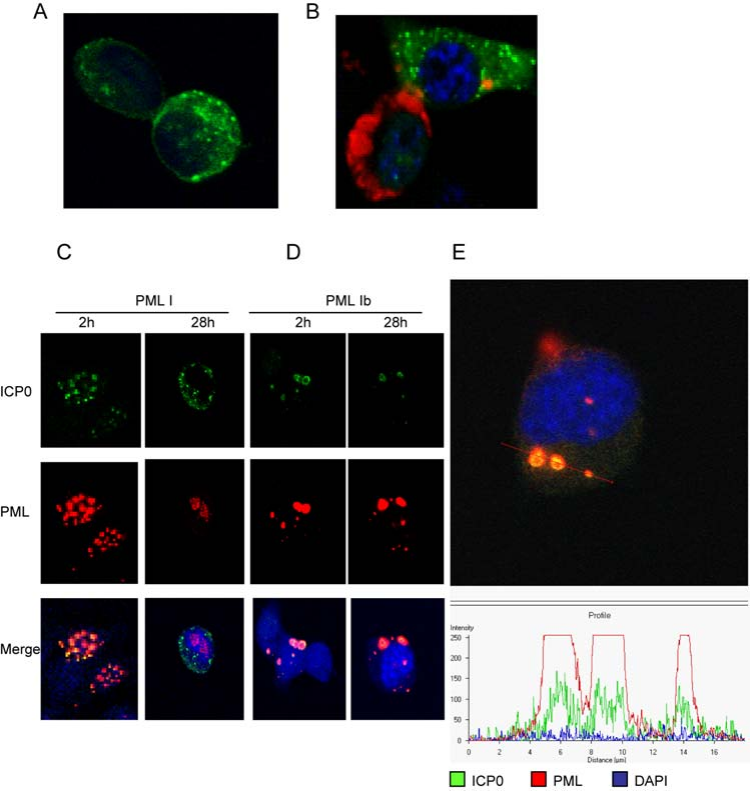
Localization of PML I or PML Ib and ICP0 in HSV-1 infected cells. (A and B). B104-1-1 cells were stably transfected with PMLIb and exposed to 0.1 PFU/cell HSV-1(F). Cells were fixed 28h post infection. Dual color immunofluorescence staining was performed with PG-M3 (diluted 1∶150) (red), and anti-ICP0 antibody (green). Chromosomal DNA was counterstained with cytox orange. Viral proteins were not detected by the PG-M3 antibody in HSV-1 infected Vector control cells (A), and ICP0 stained uniquely from PML in HSV-1 infected PML I+PML Ib co-transfected cells (compare the heavily infected cell in upper right to the recently infected cell in the lower left corner) (B). (C and D) B104-1-1 stably transfected with PML I (C) or PML Ib (D) were exposed to 0.1 PFU/cell HSV-1 (F). Cells were fixed at 2h and 28h post infection as indicated. Images were captured with a Zeiss 510 LSM confocal microscope. (E) Further analysis using the LSM Image Browser software demonstrates co-localization of PML Ib and ICP0 in the cytoplasm of a PML Ib expressing cell infected for 28 h with 0.1 PFU/cell. Red and green represent PML Ib and ICP0 respectively, while cytox orange staining is represented by blue in both the upper image and intensity profile (bottom). The intensity profile was software generated from the fluorescence intensity values encompassed along the red line (upper image).

To understand the cellular basis for the molecular interaction, we first examined the localization of PML Ib and ICP0 during HSV-1 (F) infection. Since high concentrations of the PG-M3 antibody are known cross-react with the immediate early HSV-1 protein ICP4 [43], great care was taken to use a lower concentration of antibody for these studies. Thus, fluorescence was not observed in PG-M3 stained, HSV-1(F) infected, vector control cells (Fig. 8A). In HSV-1 infected cells co-transfected with PML I+PMLIb, anti-ICP0 staining was clearly delineated from staining with the anti-PML antibody (Fig. 8B). Therefore, the staining conditions were clearly able to distinguish between PML and ICP0 proteins. As shown in Fig. 8C, cells over-expressing nuclear PML I showed a typical pattern of localization that agrees with earlier reports [41]: by 2h post infection, both ICP0 and nuclear PML co-localized at ND10. By 28h post infection, the PML I that could be visualized was dispersed diffusely but still confined in the nucleus, while ICP0 assumed a speckled pattern in the cytoplasm. In contrast, co-localization of PML Ib and ICP0 could be found throughout the course of infection in PML Ib transfected cells (Fig. 8D). Z-stack confocal analysis showed remarkable co-localization of ICP0 and PML within the cytoplasm (Fig. 8E).

**Figure 9:**
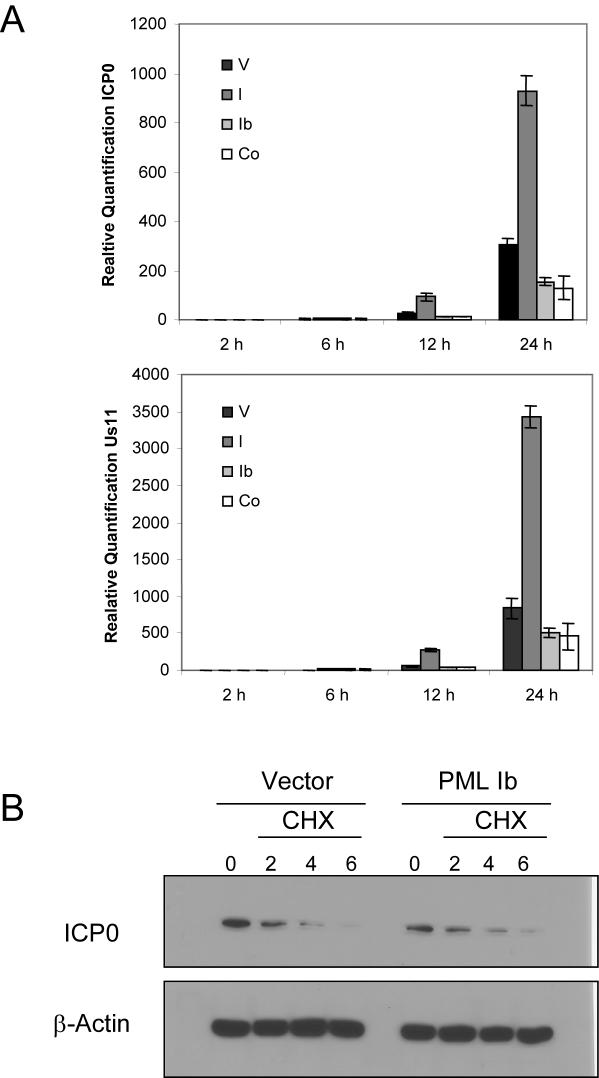
PML Ib reduces accumulation of viral transcripts but not ICP0 half-life. (A) Determination of ICP0 and Us11 expression in HSV-1 infected PML stable cell lines was determined via RT-real time-PCR. Stable cell lines containing PML I, PML Ib, PML I+PML Ib, or vector alone were seeded at 5×10^6^ cells per well in 6-well clusters and exposed to 0.01 PFU/cell of HSV-1 strain F diluted in mixture-199 (Gibco/Invitrogen) supplemented with 1% calf serum. After 1 hr incubation at 37°C, monolayers were cultured at 37°C and cells were harvested for isolation of total RNA at the indicated times. Murine 18S ribosomal RNA was used as the endogenous control. Data are representative of 2 separate trials. (B) PML Ib expression does not enhance the degradation of ICP0. Cell expressing PML Ib or vector control were exposed to 0.1 PFU/cell of HSV-1 (F). 12 h post infection, cells were treated with cyclohemimide (CHX) and protein lysates were harvested at the indicated times to monitor the expression of ICP0 by immunoblot. β-Actin was used as a loading control.

As ICP0 is a transactivator of viral gene expression in the nuclei, we reasoned that sequestration of ICP0 in the cytoplasm might be one mechanism by which PML Ib reduced expression of HSV-1 genes including ICP0. To test this hypothesis, we quantitated the amounts of ICP0 and Us11 transcripts in HSV-1 infected PML I and PML Ib cells by real-time PCR. As shown in Fig. 9A, a clear reduction of both transcripts was observed in PML Ib expressing cells 12 and 24h post infection. Conversely, PML I significantly enhanced ICP0 and Us11 transcript levels. To determine whether PMLIb affected degradation of ICP0, we treated HSV- 1 infected cells 12 hours after infection with cycloheximide and measured the amounts of protein at 2, 4 and 6 hours after the treatment. As shown in Fig. 9B, similar decay rates were observed in vector and PMLIb-transfectants. Therefore, rather than effecting the half life of ICP0 protein, it appears that PMLIb contributes to the cellular antiviral response by reducing the transcription of viral proteins via the sequestration of ICP0 in the cytoplasm.

## Discussion

It is well established that infection by a variety of viruses is associated with the disruption of PML nuclear bodies and the appearance of PML protein in the cytoplasm. It has been largely assumed that PML found in the cytoplasm of infected cells is derived from virally re-localized nuclear PML and represents a mechanism by which viruses evade the cellular defense. Here we present several lines of evidence that accumulation of cytoplasmic PML also represents a mechanism of cellular resistance to viral infection, and what was formerly interpreted as the relocation of nuclear PML is at least partially indicative of a bona fide cytoplasmic PML isoform (or isoforms) that lack a nuclear localization sequence collectively called cPMLΔ5&6.

We initially demonstrated that infection by HSV-1 (F) results in the selective enrichment of cytoplasmic isoform variants lacking exons 5 and 6, and that a novel PMLΔ5&6 variant, PML Ib is expressed during HSV infection. Although we have observed that PML Ib can be induced by IFNs, our quantitative analysis by real time PCR indicated that IFN-γ increased all PML isoforms, while HSV-1 infection favored an increased ratio of cPMLΔ5&6 to total PML. Thus, after 24 hours of IFN treatment cPMLΔ5&6 levels were not enhanced compared to total PML, but at 24 hours post infection, cPMLΔ5&6 transcripts represented about 25% of all PML transcripts. Given the large number of potential PML isoforms, it appears that cPMLΔ5&6 constitutes a major form of PML that exists during viral infection.

We subsequently demonstrated that PML Ib primarily localizes to discrete dots in the cytoplasm and near or at the nuclear membrane. PML Ib not only showed a predominant cytoplasmic localization, it also significantly effected the localization of the PMLI isoform raising the intriguing possibility that cPMLΔ5&6 may also effect the localization of predominantly nuclear PML isoforms during viral infection. Whether the shift in PML isoform transcript levels during HSV-1 infection can perpetuate the transformation in PML localization pattern remains to be determined. However, we have also observed that unlike PML isoforms I-VI, the protein product of PML Ib is not subject to modification by SUMOI (unpublished observations). Thus, is it also possible that this isoform is spared from ICP0 induced degradation of SUMOylated PML isoforms.

Interestingly, cytoplasmic PML dots similar to the staining pattern for PML Ib have been identified in the G1 phase of the cell cycle [44]. Furthermore, HSV-1 infection is known to arrest cells in G1 [45]. Thus, it will be very interesting to determine the identity of the PML isoform(s) responsible for the cytoplasmic bodies generated in G1. It is also important to note that HSV-1 infection results in the inhibition mRNA splicing early in infection largely through the action of the U_L_ 54 gene product ICP27 [46], [47]. . Like ICP0, ICP27 also regulates viral gene expression and can shuttle between the nucleus and cytoplasm [48], [49]. It is therefore of interest to test whether generation of PMLIb and other potential cPML isoforms can be mediated by ICP27, and whether or not cPML effects ICP27 function.

We next showed that the transfection of PML Ib, but not nuclear PML I, distinctly hindered viral replication. In our model system, PML Ib caused a 10 fold reduction in viral titer as well as a clear-cut reduction in the accumulation of viral proteins representative of both immediate early and late times in infection. Thus, our data helps explain a paradox regarding PML function. So far, although studies using PML ^−/−^ systems have demonstrated that PML protein is clearly part of an intrinsic cellular response against HSV-1 [33], [50], over-expression studies using nuclear PML isoforms (III , IV,V, & VI) have failed to show that any one nuclear PML isoform alone can exert such a response [24], [33], [51], [52]. In contrast, we observed that cytoplasmic PML Ib alone was capable of exhibiting an antiviral response against HSV-1. Corroborating the above findings, we found that over-expression of the predominantly nuclear localizing PML I isoform alone did not exert an antiviral function against HSV-1. In fact, we consistently observed a slight enhancement of viral proteins indicative of both immediate early and late phases of viral infection in HSV-1 infected PML I over-expressing cells. PML I is believed to be the prototypic PML isoform [41]. Our observations showed that the cellular localization pattern of this isoform in progressively infected cells mimics the localization pattern observed for viral proteins involved in stage IV HSV-1 replication compartments. Therefore, it is possible that this particular PML isoform is recruited to viral replication compartments and that a function of PML I, is exploited by the virus. How PMLI enhances viral transcription, however, remains to be determined.

We additionally demonstrated that the combination of PML I and PML Ib (which results in a cytoplasmic expression pattern) also exerts an antiviral effect. We also observed that this effect was stronger in PML ^+/+^ verses PML ^−/−^ cells suggesting the need for other PML isoforms (compare Fig. 7A and B with Fig. 5B). Overall, however, it appeared that the effect of PML Ib was dominant. In this regard, although a small minority of PML in PML I expressing cells can be found in localizing to perinuclear sites as predicted by the NES contained in exon 9 of the PML I isoform [41], [53] we showed, in co-transfection studies, that PMLI is drastically redistributed to the cytoplasm in the presence of PML Ib.. Likewise, transfected PMLIb also relocated endogenous PML from the nucleus to cytoplasm in a localization pattern consistent with previous observations [38], [54]–[56].

Since specific knock down of PML Ib enhanced viral protein accumulation in untransformed human fibroblasts, it is clear that this isoform is an intrinsic component of the cellular antiviral response. To our knowledge, this is the first report to demonstrate that a cytoplasmic PML isoform can function in antiviral defense. It is possible that the importance of cPML has been over-looked in many studies as a result of the lack of available antibodies that can successfully detect multiple endogenous PML isoforms. For example, the anti-PML antibody, 5E10, which is exceptional at recognizing multiple endogenous PML isoforms, unfortunately recognizes an epitope in exon 5 which is lacking in the cPML isoforms [57], [58]. Despite this, several studies have demonstrated an association between cellular antiviral defenses and cytoplasmically localized PML. In one study, over-expression of a cytoplasmic PML deletion mutant was capable of reducing mRNA, protein synthesis, and viral production in cells infected with influenza virus and VSV [59]. More noteworthy, human immunodeficiency virus (HIV) was shown to trigger an immediate cytoplasmic redistribution of PML that might interfere with the early steps of viral replication [23].

Lastly, our results in HSV-1 infected cells indicated that ICP0 is a target of cPML and that cPML causes the cytoplasmic sequestration of ICP0. ICP0 is an important transactivator for the transcription of viral genes including itself [31]. Our data demonstrate that PML Ib exerts its antiviral activity by decreasing viral gene expression via the cytoplasmic sequestration of ICP0 rather than by altering the half-live of ICP0.

A related possibility is that the sequestration of ICP0 via PML Ib affects the ability of ICP0 to disrupt PML NBs (ND10s) and/or to degrade nuclear PML, which has been implicated in transcriptional repression. Since the antiviral property of cPML occurs in cells without endogenous PML, our data suggest that this is not the primary mechanism by which PML Ib impairs viral growth. Yet, it has recently been demonstrated that ICP0 targets to (and subsequently disrupts) *de novo* ND10-like structures in the absence of PML [33], thus, the cytoplasmic sequestration of ICP0 mediated by PML Ib may assist the cellular antiviral defense by protecting additional ND10 resident proteins.

In summary, our data establishes a novel function for cPMLΔ5&6 in viral infection that involves the immediate early HSV-1 protein ICP0. Given the essential role of ICP0 in controlling viral gene expression and latency, an intriguing question raised by these studies is the possibility that cPMLΔ5&6 also impacts other diseases associated with recurrent viral infections. Since the disruption of PML NBs have been reported for a number of viruses, it is likely that these conclusions are not restricted to HSV-1 and are of general significance to our understanding of the cellular resistance to viral infection.

## Materials and Methods

### Cells and viruses

B104-1-1 cells, derived from NIH3T3 cells, were obtained from the American Type Culture Collection (ATCC) and cultured at 37°C in 10% CO_2_ in Dulbecco's modified Eagle's medium (DMEM) supplemented with antibiotics, glutamine and 10% calf serum. All other cell lines were cultured at 37°C in 5% CO_2_. HEK293 and MRC5 cells were obtained from ATCC and cultured according to ATCC recommendations. PML deficient (*pml* ^−/−^) mouse T antigen-immortalized fibroblasts (PML^−/−^cells) were maintained in DMEM and 10% FBS [9].

HSV-1 strain F [HSV-1 (F)] was used in these studies [60]. R7910, a gift of B. Roizman, is a recombinant virus derived from HSV-1 (F) from which both copies of the ICP0 gene have been deleted [61]. Propagation and titration of viruses were carried out using African green monkey kidney (Vero) cells (ATCC) that were maintained in DMEM plus 5% newborn calf serum (NCS).

### Antibodies

The following antibodies were used: mouse monoclonal anti-PML, PG-M3 (Santa Cruz Biotechnology), mouse monoclonal anti β-Actin (Chemicon), and antibodies to HSV-1 proteins ICP0 (1112) and gB (Goodwin Institute). Antibodies to ICP0 Exon 2 [61] and Us11 [62] were produced by B. Roizman. Polyclonal antibody to mouse PML was generated by immunization of a hamster with inclusion body from bacteria pLysBL21 containing plasmid pET32mPML which represents a full length murine PML isoform containing exon 1-9.

### Plasmid construction

Plasmid PMLVI/EBO was kindly provided by R. M. Evans. The PML VI sequence was inserted into the pcDNA3.1 Hygro vector (Invitrogen) as a Not I fragment to generate PML VI/pcDNA 3.1 Hygro. The rest of the PML plasmid constructs used in this study were generated by exchanging alternate 3′ ends representing isoforms PML I, Ib, and VI onto the 5′ sequence of PML IV corresponding to PML exons 1-3. DNA fragments specifying residues of the various carboxy-terminal regions of PML were generated as RT-PCR products that were subsequently cloned into the pNoTA/T7 (Eppendorf) or TOPO 2.1 vector (Invitrogen). The various PML constructs, ultimately housed in the pcDNA 3.1 vector using a triple ligation strategy, were designed to match the following sequences found in the NCBI data base (PML I: gi/190116/; PML Ib: gi/12275902/; PML IV:gi/12275904/; PML VI: gi/67089163/ ). Primers for plasmid construction are available upon request.

### Transfection studies

Cells were seeded 3×10^5^ cells per well of a six well cluster. Cells were transfected with 1 µg of PML Ib/pcDNA3.1 or PML I/pcDNA3.1 plasmid, both constructs, or pcDNA3.1 (vector control) using FuGene 6 according to manufacturers protocol (Roche Diagnostics Corp.). Transiently transfected cells were harvested for analysis after 48 h incubation at 37°C. Polyclonal PML ^−/−^ transfectants were cultured for two weeks in selective media containing 200ug/ml Hygromycin B, while stable clonal cell lines were generated by selecting for cells capable of growing in a limited dilution and in media supplemented with 100 or 200 µg/ml Hygromycin B (Invitrogen).

### RT-PCR and RT-real time PCR

*Analysis of endogenous PML transcripts*. MRC5 cells were treated with or without IFN-γ (1000 IU/ml, R&D systems, Inc.) for 24 h, or subject to various concentrations of HSV-1 for 24 h. Total RNA was isolated from the MRC5 cultures using Trizol reagent (Invitrogen, Carlsbad, CA) according to manufacturers instructions. The various splice forms of the PML I transcript were amplified by RT-PCR using the following primers: hPMLE3F 5′-CTTGCATCACCCAGGGGAAA3′ (sense), and hPMLE9R 5′-CCGGTTCACCGCAGCCAGCT-3′ (antisense). Real-time PCR analysis was performed using the Applied Biosystems ABI PRISM 7700 Sequence detection System with gene and isoform specific primers in the presence of Cybergreen (BioRad). Relative abundance of mRNA was calculated after normalization with GAPDH or total PML. *Analysis of viral transcripts*.Total RNA was isolated from HSV-1 infected PML stable transfectants using Trizol (Invitrogen). First strand synthesis was performed using the High capacity cDNA Reverse transcription Kit (Applied Biosystems). Real-time PCR was performed using Cyber Green (Invitrogen) and the following primer sets in conjunction with Applied Biosystem's 7500 Real Time PCR System: ICP0 F 5′ AACGCCAAGCTGGTGTACCTGATA (sence), ICP0 R 5′ AGCACCTCAAACATGCCTTTCACG (antisence), Us11 F 5′ CTTCAGATGGCTTCGAGATCGTAG (sence), Us11 R 5′ TGTTTACTTAAAAGGCGTGCCGT (antisence). Mouse 18S ribosomal RNA primer set was obtained from SuperArray.

### Indirect Immunofluorescence and confocal microscopy

Indirect immunofluorescent and confocal imagery were performed using cells cultured on 12 mm circular glass coverslips (thickness index of 1) in 6-well plates. Slide cultures were rinsed three times with PBS, fixed for 1 hour with 4% paraformaldehyde, rinsed three times with PBS, and permeablized with 0.5% Triton X-100 for 15 min. After rinsing three times with PBS, and blocking with a 5% solution of the appropriate serum diluted in PBS, cells were incubated with primary antibody overnight at 4°C (PG-M3 diluted 1∶150). Bound antibodies were detected by incubating the slide cultures with horse anti mouse Texas Red, 1∶200 for 1 h at room temperature. For dual staining of PML & ICP0, coverslips were incubated with 10% human serum, followed by incubation with anti ICPO conjugated to Alexa Fluor 488 (Molecular Probes) diluted 1∶600 in PBS. Cellular chromatin was counter stained with DRAQ5, DAPI or Cytox orange (Molecular Probes), rinsed, and mounted onto glass coverslips (AntiFade, Molecular Probes). Indirect immunofuorescent images were viewed on an Olympus fluorescence microscope and analysed using Metaview software. Confocal images were examined with a Zeiss LSM 510 confocal microscope at excitation wavelengths of 488, and 543nm. Data were collected with fourfold averaging at a resolution of 1,024×1,024 pixels.

### Immunoprecipitation

For co-immunoprecipitation of V5- and myc-tagged PML constructs, HEK293 cells were transiently transfected in 6-well plates using Fugene 6 and a total of 1 µg plasmid DNA. Cells were lysed 48 h post-transfection with IP buffer (20 mM Tris pH 8.0, 400 mM NaCl, 1 mM EDTA, 1% (v/v) Triton X-100, 10 mM NaF, 0.5% DOC, 1uM PMSF, and Protease Inhibitor Cocktail (Sigma), and protein concentrations were determined and normalized to achieve equivalent loading. Crude lysates were pre-cleared with protein G-Sepharose beads (Amersham Biosciences) and incubated with antibodies specific for V5 or myc. Immune complexes were precipitated with protein G-Sepharose beads followed by sequential washes in IP buffer. After the final wash, the pellet was resuspended in sample buffer, and proteins were resolved by SDS-PAGE (4–12% Bis-Tris NuPAGE gel) and transferred to a PDVF membrane for immunoblot analysis using HRP-conjugated V5 or Myc antibodies.

### Virus infections and growth curve experiments

Stable B1041-1 cell lines containing PML I, PMLIb or vector alone were seeded at ∼5×10^6^ cells per well in 6-well clusters and mock infected, or exposed to HSV-1 strain F or the recombinant HSV-1 ICP0 null mutant virus R7910 diluted in mixture 199 (Gibco) supplemented with 1% calf serum. After 1 hr incubation at 37°C, monolayers were rinsed extensively, and replaced with regular growth media. Cells were cultured at 37°C and harvested for titration and for western analyses at times after infection indicated in Results. For growth curve experiments, cells were subjected to two freeze/thaw cycles, sonicated and serially diluted in 199 medium containing 1% calf serum. Viral titers were determined by standard plaque assay using VERO cells.

### Preparation of cell lysates and immunoblotting

Whole cell lysates were obtained by rinsing cell monolayers once with phosphate buffered saline (PBS), and disrupting cells in 150 µl whole cell lysate buffer (20mM Tris pH8, 1mM EDTA, 1% Triton-X, 400mM NaCl, 10mM NaF, 0.5% deoxycorticosterone, 100 µM PMSF, and a protease inhibitor cocktail (Sigma). Protein concentrations were quantitated using the Bradford assay (BioRad). Equal amounts of protein lysates were denatured by boiling for 3 min in SDS loading buffer; loaded onto an 8 or 10% SDS-polyacrylamide gel; and transferred to a PVDF membrane. Membranes were blocked, and probed with the appropriate antibody overnight at 4°C (ICP0 at 1∶8000, Us11 at 1∶4000, β-Actin at 1∶5000). The immunoblots were rinsed, and incubated with secondary antibodies conjugated to horseradish peroxidase (HPR) or alkaline phosphatase for 1–3 hr at room temperature. Bound HRP conjugated antibodies were detected using enhanced chemiluminescence (ECL plus, Amersham) and bound AP conjugated antibodies were visualized by addition of the alkaline phosphatase substrate buffer containing BCIP (5-bromo 4-chloro-3-indolphosphate) and nitroblue tetrazolium (Sigma).

### RNAi

MRC5 cells (0.5×10^6^) were co-nucleofected with 100 pmol si RNA and 1 ug pUB6/V5-His (Invitrogen) using an amaxa Nucleofector Device following the manufacture's basic nucleofectin protocol for primary mammalian fibroblasts (amaxa Inc., Gaithersburg, MD). 24h after electroporation, cells were treated with 5ug/ml Blasticidin S HCl (Invitrogen) to kill all cells not harboring the blasticidin resistant gene contained in the pUB6/V5-His vector. Following 24 h of drug selection, cells were mock infected or exposed to 1 PFU/cell of the HSV-1 (F) strain or the HSV-1 ICPO-/- virus (R7910) based on a cell count taken from cells subjected to both nucleoporation and drug selection. Proteins and total RNA were harvested for Western blot and Real time PCR analysis 24 h post infection.

A pre-annealed, custom siRNA was designed to specifically target the PML Ib isoform. Both siRNA for PML Ib and a negative control siRNA were purchased from Ambion. The targeted nucleotides for PML Ib were as follows: 5′-GACGUUGACCUGAGGAACGUU-3′ (sense), 5′ CGUUCCUCAGGUCAACGUCUU-3′ (antisense).
